# Effects of thymol and carvacrol supplementation on intestinal integrity and immune responses of broiler chickens challenged with *Clostridium perfringens*

**DOI:** 10.1186/s40104-016-0079-7

**Published:** 2016-03-22

**Authors:** Encun Du, Weiwei Wang, Liping Gan, Zhui Li, Shuangshuang Guo, Yuming Guo

**Affiliations:** State Key Laboratory of Animal Nutrition, College of Animal Science and Technology, China Agricultural University, Beijing, 100193 P. R. China

**Keywords:** Broiler chicken, *Clostridium perfringens*, Essential oils, Immune response, Intestinal integrity

## Abstract

**Background:**

Necrotic enteritis caused by *Clostridium perfringens* infection leads to serious economic losses in the global poultry production. In the present study, we investigated the protective effects of essential oils (EO, which contained 25 % thymol and 25 % carvacrol as active components) supplementation on growth performance, gut lesions, intestinal morphology, and immune responses of the broiler chickens infected with *C. perfringens*. A total of 448 1-day-old male broiler chicks were allocated into eight treatment groups following a 4 × 2 factorial arrangement with four dietary EO dosages (0, 60, 120, or 240 mg/kg) and two infection status (with or without *C. perfringens* challenge from d 14 to 20).

**Results:**

The challenge did not impair the growth performance of birds, but induced gut lesions and increased crypt depth in the ileum (*P* ≤ 0.05). It also down-regulated the claudin-1 and occludin mRNA expression (*P* ≤ 0.05), up-regulated the mRNA expression of interleukin-1β (*P* ≤ 0.05), tended to increase the toll-like receptor (TLR) 2 mRNA expression (*P* < 0.10) in the ileum, and enhanced the mucosal secretory IgA production (*P* ≤ 0.05). In the challenged birds, dietary EO supplementation linearly alleviated the gut lesions and improved the ratio of villus height to crypt depth (*P ≤* 0.05), and the supplementation of 120 and 240 mg/kg EO increased the serum antibody titers against Newcastle disease virus (*P* ≤ 0.05). Regardless of challenge, the EO supplementation showed a tendency to linearly elevate the feed conversion efficiency between 14 and 28 d of age as well as the occludin mRNA expression (*P* < 0.10), and linearly inhibited the mRNA expression of TLR2 and tumor necrotic factor-α in the ileum (*P* ≤ 0.05).

**Conclusions:**

The dietary supplementation of EO could alleviate the intestinal injury by improving intestinal integrity and modulating immune responses in the *C. perfringens*-challenged broiler chickens.

## Background

Necrotic enteritis (NE) is one of the most detrimental infectious diseases in poultry, as estimated to cost the global poultry industry approximately six billion US dollars per year in medical treatments and productivity losses [1]. The aetiological agent of NE is *Clostridium perfringens*, a Gram-positive anaerobic spore-forming bacterium [2, 3]. NE may present as acute clinical or subclinical disease [2]. The acute form of NE is characterized by increased mortality in chicken flocks, whereas in the subclinical form, *C. perfringens* causes damage in the intestinal mucosa, disrupts the villus-crypt micro-architecture, decreases nutrient digestion and absorption, and thus impairs the growth performance of chickens [3, 4]. For both acute clinical and subclinical forms of NE, *C. perfringens* type A is the main pathogenic factor, and the newly discovered NetB toxin is supposed to be a vital virulence factor [3, 5].

With the removal of growth-promoting antibiotics, NE has become increasingly prevalent [3], and this problem has inspired the research in alternative managements and dietary strategies to control the incidence and severity of NE [4]. Due to the consumer preference for natural products, the application of essential oils (EO) have been increasing in appeal [6]. The antibacterial property of EO has been well recognized and widely tested in vitro against a wide range of pathogenic bacteria, including both Gram-positive and Gram-negative bacteria [7–10].

Besides direct antibacterial effects, the dietary EO supplementation has been reported to improve intestinal integrity and strengthen mucosal barrier [11, 12]. In broiler chickens, the addition of 0.5 g/kg thyme oil could increase the trans-epithelial electrical resistance of the duodenum [12]. The mice receiving 13.3 μg/mL eugenol in drinking water altered the composition of colonic microbiota and increased the thickness of the inner mucus [11]. Furthermore, the EO supplementation has been reported to enhance the cellular and humoral immunity [13, 14], and modulate the gene expression of host immunity in chickens [15]. Among thousands of EO constituents, the two isomers thymol and carvacrol possess great antibacterial activity, which are also major components of common used herbs such as thyme and oregano [16]. In rodents, thymol and carvacrol have been reported to inhibit pro-inflammatory cytokines,decrease inflammatory cell recruitment and alleviate oxidative damage [17–19]. Our previous study suggested that a mixture of thymol and carvacrol could decrease the tumor necrotic factor (TNF)-α gene expression and increase the interleukin (IL)-4 gene expression in the spleen of the broilers injected with lipopolysaccharides (LPS) [20]. Therefore, we hypothesized that the supplementation of EO may protect birds from NE through their modulatory effects on intestinal integrity and immunity.

## Methods

### Birds and experimental design

A total of 448 1-day-old male broiler chicks (Cobb 500) were used in a 28-day experiment. Chicks were allocated into eight treatment groups and each group consisted of eight replicate pens (seven birds each pen). The experiment was following a 4 × 2 factorial arrangement in a randomized complete block design to evaluate the effects of dietary EO dosages, pathogen challenge and their interaction, as shown in Table 1. Birds in groups U1, U2, U3 and U4 were unchallenged and fed the wheat-based diet supplemented with 0, 60, 120, or 240 mg/kg EO, respectively. Correspondingly, birds in groups C1, C2, C3, and C4 were challenged with *C. perfringens* and fed the wheat-based diet supplemented with 0, 60, 120, or 240 mg/kg EO, respectively. The EO preparation used in this trial was a commercial product provided by Novus International Inc. (St Charles, MO, USA), which contained 25 % thymol and 25 % carvacrol as active components, 37 % silicon dioxide as caking inhibitor, and 13 % glycerides as stabilizing agents.

**Table 1:** Experimental design^a^

Treatments	Dietary EO dosage (mg/kg)	*C. perfringens* challenge
U1	0	Unchallenged
U2	60	Unchallenged
U3	120	Unchallenged
U4	240	Unchallenged
C1	0	Challenged
C2	60	Challenged
C3	120	Challenged
C4	240	Challenged

^a^ *EO* essential oils; Unchallenged, birds without oral gavage of *C. perfringens*; Challenged, birds with oral gavage of 1.0 mL actively growing culture of *C. perfringens* from d 14 to d 20 (1.0 × 10^8^ cfu/mL)

### Diet formulation

Chickens were fed starter (d 0–21) and grower (d 22–28) diets in the form of mash. The basal diets were formulated to meet or exceed the feeding standard of China for broilers (NY/T 2004) [21] and the diet composition was shown in Table 2. To promote the proliferation of *C. perfringens*, the antibiotic-free and coccidiostat-free wheat-based diets were formulated. Both wheat and soybean meal were pre-mixed in advance and the basal diet was subsequently mixed to reduce mixing variation among treatments. Then, the EO preparation was carefully added to 100 g of the basal diet, and blended with 10 kg of the basal diet to make a premix. Finally, the premix was mixed in the basal diet. All of the experimental diets were manufactured weekly at the feed mill of China Agricultural University, and stored in airtight containers.

**Table 2:** Diet composition and nutrient levels

Items (%, unless otherwise indicated)	Starter diets (d 0–21)	Grower diets (d 22–28)
Ingredient		
Wheat	62.75	68.50
Soybean meal	29.61	23.72
Soybean oil	3.40	4.00
Dicalcium phosphate	1.91	1.63
Limestone	1.04	0.96
Sodium chloride	0.35	0.35
Choline chloride (50 %)	0.25	0.25
L-Lysine (99 %)	0.25	0.24
DL-Methionine (98 %)	0.19	0.11
Santoquin	0.03	0.03
Trace mineral premix^a^	0.20	0.20
Vitamin premix^b^	0.02	0.02
Calculated nutrient level		
ME (Mcal/kg)	2.90	2.98
Protein	21.00	19.00
Calcium	1.00	0.90
Available Phosphorus	0.45	0.40
Lysine	1.15	1.00
Methionine	0.50	0.40

^a^The trace mineral premix provided the following (per kg of diet): manganese, 100 mg; zinc, 75 mg; iron, 80 mg; copper, 8 mg; selenium, 0.25 mg; iodine, 0.35 mg

^b^The vitamin premix provided the following (per kg of diet): vitamin A, 18,750 IU; vitamin D_3_, 3750 IU; vitamin E, 28 IU; vitamin K_3_, 3.975 mg; thiamine mononitrate, 3 mg; riboflavin, 9 mg; vitamin B_12_, 0.0375 mg; d-biotin, 0.150 mg; folic acid, 1.875 mg; d-calcium pantothenate, 18 mg; nicotinic acid, 75 mg

### Animal husbandry

Approval for this study was obtained from the Animal Ethics Committees of China Agricultural University. Chickens were housed in the battery pens (100 cm × 70 cm) with plastic wire floors and two nipple-type waterers each pen. Feed and water were provided ad libitum throughout the experiment. The room temperature was maintained at 36 °C for the first week and then reduced by 3 °C each week until reaching 24 °C. The lighting schedule was 23 h light and 1 h dark throughout the experiment. In addition, the chickens were vaccinated against Newcastle disease virus (NDV) after hatch and on d 10 according to the routine immunization programme.

### *Clostridium perfringens* challenge and sampling

*C. perfringens* challenge was based on the method originally developed by Dahiya et al. [22], and also previously used in our lab [23, 24]. The *C. perfringens* strain we used was type A field strain, which was isolated from a clinical case of NE and did not carry the NetB gene as determined by polymerase chain reaction (PCR). Briefly, the organism was cultured anaerobically on tryptose-sulphite-cycloserine for 18 h at 37 °C, and then aseptically inoculated into cooked meat medium and incubated anaerobically overnight at 37 °C. All birds in the challenged groups were orally gavaged in the crop with 1.0 mL actively growing culture of *C. perfringens* each day from d 14 to d 20 (1.0 × 10^8^ cfu/mL). Birds in the unchallenged groups received the same volume of sterile meat medium. On d 21, one bird from each replicate was randomly selected and killed by intracardial administration of sodium pentobarbital (30 mg per kg of body weight) and jugular exsanguination for tissue sampling.

### Analysis of dietary thymol and carvacrol concentrations

To extract thymol and carvacrol from the diets, 4 g of grinded diets were weighted into centrifuge tubes, mixed with 2.5 mL water and 1.0 mL ethanol, and allowed to stand for 15 min. The calibration samples were prepared by supplemented the basal diet with standard solutions of thymol and carvacrol at five different concentrations (300, 150, 75, 37.5 and 18.75 mg/L in ethanol). Both thymol and carvacrol were purchased from Sigma-Aldrich Corporation (St Louis, MO, USA) with compound purities at ~ 98 %. Then diethyl ether (12 mL) was added, and the samples were shaken for 16 h and centrifuged at 15, 000 g for 5 min. 1 μL of each supernatant was used for gas chromatographic analysis according to the method of Michiels et al. [25].

### Growth performance

Feed intake (FI) and body weight (BW) for each replicate were measured on d 14 and 28. Feed conversion ratio (FCR) was calculated during d 0 to14 and d 14 to 28.

### Intestinal lesion score

The small intestine from each bird was opened and scored blindly by three independent observers as described by Dahiya et al. [22]. Briefly, lesions were scored using a scale from 0 to 4, in which 0 was apparently normal intestinal appearance, no lesion; 0.5 = severely congested serosa and mesentery engorged with blood; 1 = thin walled and friable intestines with small red petechiae (>5); 2 = focal necrotic lesions; 3 = patches of necrosis (1 to 2 cm long); and 4 = diffused necrosis typical of field cases.

### Intestinal morphological analyses

The distal ileum segments were collected and fixed in 4 % paraformaldehyde and then embedded in paraffin. Tissue sections (5 μm) were prepared and stained with haematoxylin and eosin for the morphological analyses. The measurements were performed with an Olympus optical microscope using ProgRes CapturePro software (version 2.7; Jenoptik, Jena, Germany). Nine villi were measured for each section and only complete and vertically oriented villi were measured. Villus height was measured from the tip of the villus to the crypt opening and the associate crypt depth was measured from the base of the crypt to the level of the crypt opening. Then the ratio of villus height to relative crypt depth (V:C ratio) was calculated from these measurements.

### Serum and mucosal antibody levels

On d 21, blood was aseptically collected from the wing vein into vacutainers and centrifuged at 3,000 rpm for 15 min to obtain the serum. The serum antibody titers against NDV were determined by the commercial ELISA kit (IDEXX laboratories Inc., Westbrook, Maine, USA) according to the manufacture’s protocol. Intestinal mucosa was scraped from 10 cm of the jejunum (proximal to Meckel’s diverticulum) and homogenized with phosphate buffered saline. The secretory IgA (sIgA) concentrations and protein content in mucosal homogenates were determined by ELISA kit (Bethyl Laboratories Inc., Montgomery, TX, USA) and BCA protein assay kit (Thermo Scientific) according to the manufacture’s protocol, respectively. The final sIgA concentrations were expressed as mg/g protein.

### Lymphocytes proliferative responses

Blood was collected into the vacutainers containing heparin to prevent clotting on d 21. Then peripheral blood mononuclear cells (PBMC) were isolated using Ficoll density centrifugation according to the method of Tan et al. [26] with some modifications. Briefly, heparinized blood was diluted with Hank’s balanced salt solution by 1:1 (no calcium, no magnesium, Life Technologies), and layered on the top of Histopaque 1077 (Sigma-Aldrich Corporation) carefully in a 10 mL centrifuge tube (2:1). After centrifugation for 30 min at 3,000 rpm (20 °C), the PBMC at the plasma-ficoll interface were collected. Then PBMC was washed three times with cold RPMI-1640 medium (containing 5.0 % inactivated fetal bovine serum, 0.0599 mg/mL penicillin, 100 μg/ml streptomycin and 24 mM-HEPES) by centrifugation at 1,800 rpm for 10 min (4 °C). Cell counts and viability were evaluated using trypan blue staining. The proliferative response of T cells and B cells after stimulation with concanavalin A (ConA, 45 μg/mL) and LPS (25 μg/mL) were determined by MTT assay, respectively, as described by Wagner et al. [27]. ConA from *Canavalia ensiformis* (C2010) and LPS from *Escherichia coli* (L2880) were both obtained from Sigma-Aldrich Corporation. Results were expressed as stimulation index (SI).

### Total RNA extraction and reverse transcription

Total RNA was extracted from intestinal segments using Trizol reagent (Invitrogen Life Technologies, Carlsbad, California, USA) following the manufacturer’s protocol. The concentration of extracted RNA was measured using a NanoDrop spectrophotometer (ND-1000, NanoDrop Products, Wilmington, Delaware, USA) at an optical density of 260 nm, and RNA purity was verified by the ratio of absorbance at 260 nm/280 nm. Then, 1 μg of total RNA was used for reverse transcription by a reverse transcription kit (Takara Bio Inc., Dalian, China) following the manufacturer’s protocol. All the cDNA preparations were stored at −20 °C until further use.

### Real-time quantitative PCR

Expression levels of the following genes were analyzed by real-time quantitative PCR (RT-PCR): toll-like receptor (TLR) 2, TLR4, IL-1β, TNF-α, claudin-1, occludin, mucin-2 and an endogenous reference gene β-actin (which has been previously used [24, 28]). Gene-specific primer sequences are shown in Table 3. The RT-PCR was performed on the 7500-fluorescence detection system (Applied Biosystems, Foster City, California, USA) using a commercial SYBR-Green PCR kit (Takara Bio Inc.). According to the manufacturer’s protocol, the following PCR conditions were employed: 95 °C for 30 s, 40 cycles of 95 °C for 5 s and 60 °C for 34 s, and followed by the stage of melting curve. At the end of each run, melting curve analysis and subsequent agarose gel electrophoresis of the PCR products were subjected to confirm the amplification specificity. Relative gene expression data were analyzed using the 2^−ΔΔCt^ method as developed by Livak et al. [29].

**Table 3:** RT-PCR primers and Genbank accession numbers of chicken

Target	Primer sequence	Accession no.	Product size, bp
	(5′–3′)^a^		
TLR2	F: GATTGTGGACAACATCATTGACTC	NM_001161650	294
	R: AGAGCTGCTTTCAAGTTTTCCC		
TLR4	F: AGTCTGAAATTGCTGAGCTCAAAT	NM_001030693	190
	R: GCGACGTTAAGCCATGGAAG		
IL-1β	F: ACTGGGCATCAAGGGCTA	NM_204524	131
	R: GGTAGAAGATGAAGCGGGTC		
TNF-α	F: GAGCGTTGACTTGGCTGTC	NM_204267	64
	R: AAGCAACAACCAGCTATGCAC		
Claudin-1	F: CATACTCCTGGGTCTGGTTGGT	AY750897.1	100
	R: GACAGCCATCCGCATCTTCT		
Occludin	F: ACGGCAGCACCTACCTCAA	D21837.1	123
	R: GGGCGAAGAAGCAGATGAG		
Mucin-2	F: TTCATGATGCCTGCTCTTGTG	XM_421035	93
	R: CCTGAGCCTTGGTACATTCTTGT		
β-actin	F: GAGAAATTGTGCGTGACATCA	L08165	152
	R: CCTGAACCTCTCATTGCCA		

^a^ *F* means forward, *R* means reverse

### Statistical analyses

Results are given as mean values and pooled standard errors. Data were analyzed using the general linear model procedure of SAS software (SAS Institute Inc., Cary, NC, USA), and subjected to two-way ANOVA in a 4 × 2 factorial arrangement to analyze the main effects of EO supplementation and pathogen challenge, and their interaction. When the interaction was significant, polynomial contrasts were performed with the individual means in the unchallenged groups and challenged groups respectively, and the linear and quadratic responses to EO dosages were determined. If the interaction was not significant, polynomial contrasts were conducted to determine the linear and quadratic responses of the main-effect means (averaged between the unchallenged and challenged groups) to dietary EO dosages. Statistical significance was set at *P* ≤ 0.05, and 0.05 < *P* < 0.10 was considered a trend towards significance.

## Results

### Dietary thymol and carvacrol concentrations

The intended concentrations of thymol and carvacrol were 15, 30, and 60 mg/kg for the diets supplemented with 60, 120, and 240 mg/kg EO, respectively (Table 4). According to the quantitative analysis, the thymol concentrations were 94.5–95.1 % of the desired amount, and the carvacrol concentrations were 101.1–103.1 % of the desired amount. None of thymol or carvacrol was detected in the unsupplemented basal diet.

**Table 4:** Intended and analyzed concentrations of thymol and carvacrol in the experimental diets

Item	Dietary EO concentration, mg/kg			
	0	60	120	240
Thymol	ND	15.0/14.2^a^	30.0/28.5	60.0/56.8
Carvacrol	ND	15.0/15.2	30.0/30.9	60.0/61.9

*ND* not detected. ^a^Intended concentration in the diet/analyzed concentration in the diet

### Growth performance

Dietary supplementation of 60, 120 and 240 mg/kg EO did not influence the growth performance of the broilers during d 0 to 14 (data not shown). BW gain (BWG) and FI were not impaired by *C. perfringens* challenge (Table 5). Between 14 and 28 d of age, the EO addition tended to linearly reduce the FCR (*P* = 0.056). The relative weight of spleen was increased due to *C. perfringens* challenge on d 21 (*P* = 0.004). No interaction on the growth performance was found between EO addition and *C. perfringens* challenge (*P* > 0.10).

**Table 5:** Effect of essential oils on the growth performance and relative spleen weight of broilers^a^

Items	BWG (d 14–28), g	FI (d 14–28), g	FCR (d 14–28)	Spleen (d 21), %
Treatment				
U1	810	1435	1.77	0.10
U2	833	1407	1.70	0.11
U3	859	1454	1.70	0.12
U4	868	1436	1.66	0.11
C1	883	1510	1.71	0.11
C2	878	1480	1.69	0.14
C3	827	1395	1.69	0.13
C4	836	1418	1.70	0.14
SEM	11.2	14.3	0.01	0.004
*P*-value				
Challenge	0.559	0.553	0.569	0.004
EO	0.984	0.617	0.148	0.289
Challenge × EO	0.261	0.262	0.392	0.426
Linear^b^	0.938	0.288	0.056	0.155
Quadratic^b^	0.925	0.434	0.305	0.338

^a^Values are means of eight replicates per treatment. *BWG* body weight gain, *FI* feed intake, *FCR* feed conversion ratio, *SEM* pooled standard error, *EO* essential oils

^b^When the interaction between *C. perfringens* challenge and EO supplementation was not significant, linear and quadratic polynomial contrasts were performed on the main-effect means to EO dosages

### Intestinal lesion score

No intestinal lesions were observed in the unchallenged birds. In the challenged birds, intestinal lesion scores were linearly reduced with increasing EO dosages (*P* = 0.010, Table 6), and the lesion severity of broilers in group C4 was milder than that of birds in group C1 (*P* ≤ 0.05). However, *C. perfringens* challenge did not lead to mortality in the present study.

**Table 6:** Effect of essential oils on the intestinal lesion score of the *Clostridium perfringens*-challenged broilers ^c^

Item	C1	C2	C3	C4	SEM	*P*-value	Linear^d^	Quadratic^d^
Lesion score	1.50^a^	0.93^ab^	0.71^ab^	0.29^b^	0.166	0.065	0.01	0.514

^a,b^Mean values within the same row not sharing a common uppercase superscript letter differ significantly (*P* ≤ 0.05)

^c^Values are means of eight replicates per treatment. *SEM* pooled standard error

^d^Linear and quadratic polynomial contrasts to EO dosages

### Intestinal morphology

The challenge of *C. perfringens* significantly increased crypt depth in the ileum (*P* = 0.005, Table 7). Although the dietary addition of 60–240 mg/kg EO did not influence villus height or crypt depth, the pathogen challenge and EO supplementation showed a significant interactive effect on the V:C ratio (*P* = 0.003). In the challenged birds, the EO supplementation linearly and quadratically increased the V:C ratio (*P* ≤ 0.05), and the ratio was greatly increased by the EO-included diets compared with the basal diet (*P* ≤ 0.05). The thickness of the muscular layer was not significantly influenced by *C. perfringens* challenge or EO supplementation.

**Table 7:** Effect of essential oils on the ileal morphology of broilers^c^

Items	VH, μm	CD, μm	V:C	ML, μm
Treatment				
U1	442.8	194.3	2.32	201.6
U2	395.2	206.6	2.12	201.8
U3	376.0	225.8	1.74	189.4
U4	488.7	214.7	1.98	181.2
C1	412.7	270.0	1.32^b, *^	159.2
C2	472.0	245.0	1.95^a, *^	160.8
C3	552.3	270.2	1.93^a, *^	184.3
C4	451.0	237.3	1.99^a, *^	201.7
SEM	18.69	7.94	0.06	5.93
*P*-value				
Challenge	0.220	0.005	0.035	0.161
EO	0.805	0.712	0.450	0.898
Challenge × EO	0.151	0.668	0.003	0.186
Linear ^d^	0.370	0.664	NA	0.371
Quadratic ^d^	0.668	0.687	NA	0.985

^a,b^Mean values within the same column not sharing a common uppercase superscript letter differ significantly (*P* ≤ 0.05)

^c^Values are means of eight replicates per treatment. *VH* villus height, *CD* crypt depth, *V:C* the ratio of villus height to crypt depth, *ML* thickness of muscular layer, *SEM* pooled standard error, *EO* essential oils, *NA* not analyzed

^d^When the interaction between *C. perfringens* challenge and EO supplementation was not significant, linear and quadratic polynomial contrasts were performed on the main-effect means to EO dosages

^*^ When the interaction was significant, mean values within the challenged or unchallenged groups have a linear and quadratic dose response to EO dosages (*P* ≤ 0.05)

### Gene expression of tight junction proteins and mucin-2

According to Table 8, *C. perfringens* challenge decreased the claudin-1 and occludin gene expression in the ileum (*P* = 0.006 and 0.031, respectively). The occludin gene expression tended to be up-regulated linearly with increasing EO dosages (*P* = 0.099). The expression of mucin-2 was not significantly influenced by *C. perfringens* challenge or EO addition.

**Table 8:** Effect of essential oils on the ileal gene expression of claudin-1, occludin and mucin-2 in broilers^a^

Item	Claudin-1	Occludin	Mucin-2
Treatment			
U1	1.04	1.01	1.05
U2	0.99	0.84	0.63
U3	1.27	1.11	0.95
U4	1.33	1.09	0.82
C1	0.98	0.80	0.86
C2	0.95	0.91	0.80
C3	0.80	0.93	0.68
C4	0.80	0.94	0.93
SEM	0.051	0.029	0.047
*P*-value			
Challenge	0.006	0.031	0.627
EO	0.908	0.130	0.335
Challenge × EO	0.136	0.250	0.281
Linear^b^	0.599	0.099	0.892
Quadratic^b^	0.894	0.766	0.159

^a^Values are means of eight replicates per treatment. *SEM* pooled standard error, *EO* essential oils

^b^When the interaction between *C. perfringens* challenge and EO supplementation was not significant, linear and quadratic polynomial contrasts were performed on the main-effect means to EO dosages

### Inflammation-related gene expression

*C. perfringens* challenge enhanced the gene expression of TLR2 and IL-1β in the ileum (*P* = 0.057 and 0.042, respectively, Table 9), but did not significantly influence the gene expression of TLR4 and TNF-α. Dietary EO supplementation linearly (*P* = 0.050) and quadratically (*P* = 0.023) down-regulated the TLR2 gene expression, and linearly decreased the TNF-α gene expression (*P* = 0.020).

**Table 9:** Effect of essential oils on the ileal inflammation-related gene expression of broilers^a^

Item	TLR2	TLR4	TNF-α	IL-1β
Treatment				
U1	1.28	1.02	1.03	1.09
U2	1.18	1.05	0.84	1.34
U3	0.74	0.97	0.92	1.00
U4	1.10	0.93	0.82	1.08
C1	1.57	1.10	1.07	1.74
C2	1.36	0.97	0.90	1.52
C3	1.08	1.02	1.06	1.53
C4	1.15	0.88	0.85	1.27
SEM	0.061	0.043	0.028	0.091
*P*-value				
Challenge	0.057	0.963	0.193	0.042
EO	0.015	0.669	0.018	0.722
Challenge × EO	0.777	0.902	0.860	0.707
Linear^b^	0.050	0.204	0.020	0.261
Quadratic^b^	0.023	0.954	0.851	0.949

^a^Values are means of eight replicates per treatment. *SEM* pooled standard error, *EO* essential oils

^b^When the interaction between *C. perfringens* challenge and EO supplementation was not significant, linear and quadratic polynomial contrasts were performed on the main-effect means to EO dosages

### Humoral immunity and cellular immunity parameters

A significant interaction on serum NDV antibody titers was observed between *C. perfringens* challenge and the EO supplementation (*P* < 0.001, Table 10). In the challenged birds, the NDV antibody titers were linearly and qudratically elevated with increasing EO dosages (*P* ≤ 0.05). To be specific, the NDV antibody titers of birds in groups C3 and C4 were higher than those of birds in groups C1 and C2 (*P* ≤ 0.05), and the highest antibody titers were found in group C4 among all challenged groups (*P* ≤ 0.05). However, no significant differences in the NDV antibody titers were found in the unchallenged groups. In addition, *C. perfringens* challenge greatly increased the mucosal sIgA levels (*P* ≤ 0.05), which was not significantly affected by the EO supplementation. No remarkable differences were found in the proliferative responses of T cells and B cells, as evidenced by unchanged ConA and LPS SI in vitro.

**Table 10:** Effect of essential oils on the humoral and cellular immunity parameters of broilers^d^

Item	NDV antibody titers, log_10_	sIgA, mg/g protein	ConA SI	LPS SI
Treatment				
U1	3.01	2.24	1.14	1.12
U2	2.87	2.16	1.04	0.94
U3	2.96	2.24	0.97	1.12
U4	2.79	2.51	0.94	1.11
C1	2.80^c, *^	3.84	1.05	1.20
C2	3.14^c, *^	3.43	0.82	1.04
C3	4.13^b, *^	3.42	0.99	1.01
C4	4.24^a, *^	3.22	0.94	1.00
SEM	0.085	0.259	0.031	0.03
*P*-value				
Challenge	<0.001	0.033	0.217	0.836
EO	<0.001	0.991	0.183	0.279
Challenge × EO	<0.001	0.950	0.505	0.425
Linear^e^	NA	0.800	0.157	0.493
Quadratic^e^	NA	0.760	0.255	0.225

^a,b,c^Mean values within the same column not sharing a common uppercase superscript letter differ significantly (*P* ≤ 0.05)

^d^Values are means of eight replicates per treatment. *NDV* Newcastle disease virus, *sIgA* secretory IgA, *ConA* concanavalin A, *LPS* lipopolysaccharides, *SI* stimulation index, *SEM* pooled standard error, *EO* essential oils, *NA* not analyzed

^e^When the interaction between *C. perfringens* challenge and EO supplementation was not significant, linear and quadratic polynomial contrasts were performed on the main-effect means to EO dosages

^*^ When the interaction was significant, mean values within the challenged or unchallenged groups have a linear and quadratic dose response to EO dosages (*P* ≤ 0.05)

## Discussion

In the present study, the wheat-based diets and multiple oral gavages of *C. perfringens* were used to induce the experimental NE. High levels of soluble non-starch polysaccharides in the wheat could favor the proliferation of intestinal *C. perfringens* and cause more gut lesions [2, 30, 31]. However, the challenge did not result in overtly clinical signs of NE or NE-related mortality in the present study. The deficiency of the NetB gene in the *C. perfringens* strain we used might partially explain the absence of characteristic NE, since NetB has been demonstrated to play a major role in the pathogenesis of NE [3, 5]. Despite this, *C. perfringens* challenge damaged the intestinal mucosa, as observed by macroscopic lesions, which was similar to previous studies using the same challenge model to create the sub-clinical NE [23, 24]. In the current study, dietary supplementation of thymol and carvacrol-bearing EO alleviated the intestinal lesions, increased the intestinal V:C ratio, decreased the inflammatory response and improved the specific antibody titers in the challenged birds. These results suggested that the EO preparation had protective effects against *C. perfringens* challenge. And this might, at least partially, be associated with their modulation on intestinal integrity and immunity.

Usually, damaged mucosa caused by *C. perfringens* leads to retarded growth rate and poor feed conversion efficiency in chickens [3, 4]. However, FI and BWG were not significantly influenced in the present study, which was similar to the results obtained by Grilli et al. [32]. In the current study, the EO supplementation did not significantly influence the growth performance except that FCR tended to be reduced with increasing EO dosages during 14 to 28 days of age. In fact, the effects of EO on the growth performance of piglets and poultry were variable. However, the majority of experimental results indicated reduced FI at largely unchanged BWG or final BW, which led to an improved feed conversion ratio when feeding EO [6, 33, 34]. Generally speaking, the stimulation of digestive secretions (e.g., saliva and bile) and enhanced enzyme activity were proposed to be a core mode of EO to improve the feed conversion efficiency [33–36].

In the current study, dietary EO supplementation linearly decreased macroscopic gut lesions, and improved intestinal histomorphology microscopically in the challenged birds. Morphology of the small intestine can be used as an indicator for intestinal health and integrity [37]. Deeper crypts indicate faster cellular turnover to permit renewal of the villus as needed in response to normal sloughing or inflammation induced by pathogens or their toxins [37]. In this study, *C. perfringens* challenge led to remarkably deeper crypt in the ileum and pronounced intestinal lesions. Similar observations were reported in birds infected with *C. perfringens* [24, 37]. In the challenged birds, however, the dietary EO supplementation linearly alleviated the intestinal lesions, and 60–240 mg/kg EO numerically increased the villus height and decreased the crypt depth, which resulted in significantly elevated V:C ratio, indicating mature enterocytes and efficient ability of nutrient absorption. The beneficial effects of EO on intestinal lesions and histomorphology might be associated with their antibacterial activity and stabilizing effects on intestinal microflora [33, 34]. Also, the anti-inflammatory effects of EO might favor the intestinal health and develop better villus-crypt micro-architecture.

The intestinal mucosa is not only the major site for nutrient digestion and absorption, but also plays a key role in host defense against pathogen infection. Tight junction (TJ) proteins form a dynamic seal between epithelial cells and act as a fence preventing macromolecular transmission. The disruption of intestinal TJ proteins could lead to increasing permeability to luminal antigens and bacteria translocation [38]. However, few data are available regarding TJ protein expression in *C. perfringens*-infected birds, except that Liu et al. reported decreased occludin gene expression in the small intestine of the infected birds [24]. In the present study, decreased ileal gene expression of occludin and claudin-1 was also observed in the challenged broilers, and the dietary supplementation of EO tended to linearly increase the occludin gene expression. Besides TJ proteins, mucus layer is the first barrier of defense encountered by intestinal bacteria, and mucins are the primary constituent of the mucus layer [39]. Mucin-2, the major mucin gene in the small intestine, was not significantly influenced by *C. perfringens* infection in the present study, which was similar to previous studies [24, 39]. And EO supplementation also did not affect the gene expression of mucin-2.

During evolution, pattern recognition receptors (PRRs) are selected to recognize the conserved components of microorganisms or pathogen associated molecular patterns, including LPS and peptidoglycans [40]. TLRs are important members of PRRs, which trigger subsequent inflammatory responses through MyD88 dependent or independent signaling pathways and finally lead to release of pro-inflammatory cytokines [40, 41]. In mammals, TLR4 could recognize LPS, which is unique to Gram-negative bacteria, and TLR2 could recognize peptidoglycans, which is abundant in Gram-positive bacteria [41]. In the present study, the intestinal TLR2 gene expression was up-regulated in the *C. perfringens-*challenged birds, but the TLR4 gene expression was not significantly affected. Similar results were obtained by Cao et al. [28]. Of note, however, some investigators reported no obvious alteration of the TLR2 gene expression was observed in chicken intestine and primary intestinal epithelial cells [42, 43]. The controversial results might be related with dosage and frequency of *C. perfringens* infection, which need further investigation.

TNF-α and IL-1β are the important pro-inflammatory cytokines, which regulate the host immunity against multiple pathogens through immune cell differentiation, proliferation, apoptosis and NO production [44]. However, excessive and long-term production of pro-inflammatory cytokines might lead to gut damage and high consumption of energy [44]. Thus, the suppression of TLR2 and pro-inflammatory cytokine TNF-α by the dietary EO supplementation in the current study might alleviate inflammation and improve gut health, as evidenced by attenuated gut lesions and increased V:C ratio in the ileum. Actually, anti-inflammatory effects of thymol and carvacrol have been well documented in pharmacological experiments with rodents. They could inhibit pro-inflammatory cytokines,decrease inflammatory cell recruitment, alleviate oxidative damage and thus reduce tissue injury [17–19]. However, limited information is available about the effects of EO on the production of cytokines and chemokines in poultry. One of our previous studies demonstrated that 60 mg/kg of the same EO preparation showed anti-inflammatory effects by decreasing the TNF-α gene expression and increasing the IL-4 gene expression in the broilers challenged with multiple LPS injections [20]. Besides the important roles in immunity, cytokines were also demonstrated to affect TJ. Pro-inflammatory cytokines could induce disruption of TJ, which led to increased intestinal permeability, whereas anti-inflammatory cytokines tended to protect the intestinal integrity [45]. In the present study, dietary EO supplementation suppressed the TNF-α gene expression, which was in accordance with the trend of increased occludin gene expression in the ileum.

To explore the effects of EO on humoral immunity, specific antibody titers were analyzed. We observed that dietary supplementation of 120 and 240 mg/kg EO significantly increased the serum NDV antibody titers compared with the basal diet or diet with 60 mg/kg EO in the challenged birds. This was consistent with previous studies, which demonstrated that dietary supplementation of EO enhanced the specific immune responses in broilers and laying hens [14, 46, 47]. As the most abundant immunoglobulin isotype in mucosal secretions, sIgA is the fundamental effector to mucosal immunity. In the present study, *C. perfringens* challenge induced sIgA secretion, which was not significantly affected by the EO supplementation. In addition to humoral immunity, 5 mg/kg carvacrol-bearing EO and 707 mg/kg carvacrol-bearing oregano oil was reported to promote the lymphocyte proliferation [13, 48]. Under certain concentrations, however, carvacrol could suppress the porcine lymphocyte proliferation, which might occur through the induction of apoptosis [49]. We did not found any remarkable effect of *C. perfringens* challenge or EO supplementation on the proliferative responses of T cells and B cells in vitro.

## Conclusions

In conclusion, the dietary supplementation of 60, 120 and 240 mg/kg EO (which contained 25 % thymol and 25 % carvacrol as active components) alleviated the intestinal lesions, improved the intestinal histomorphology, decreased the inflammatory response and enhanced the specific immune response in the *C. perfringens*-challenged broiler chickens. Based on the results obtained in this study, 240 mg/kg of the EO preparation was an optimum supplementation dose for protecting broiler chickens against *C. perfringens* challenge. The beneficial effects of EO might, at least partially, be associated with their modulatory effects on the intestinal integrity and immunity. More studies are needed to further define the underlying molecular mechanisms.

## Abbreviations

BW: body weight
BWG: body weight gain
ConA: concanavalin A
d: day
EO: essential oils
FCR: feed conversion ratio
FI: feed intake
IL: interleukin
LPS: lipopolysaccharides
NDV: Newcastle disease virus
NE: necrotic enteritis
PBMC: peripheral blood mononuclear cells
PCR: polymerase chain reaction
PRRs: pattern recognition receptors
RT-PCR: real-time quantitative PCR
SI: stimulation index
sIgA: secretory IgA
TJ: tight junction
TLR: toll-like receptor
TNF: tumor necrotic factor
V:C ratio: the ratio of villus height to crypt depth
